# Photodynamic Therapy for Obstructive Esophageal Malignancies

**DOI:** 10.1155/DTE.5.167

**Published:** 1999

**Authors:** James S. McCaughan

**Affiliations:** Grant Medical Center 323 E. Town Street Columbus OH 43215 USA

## Abstract

*Objectives* Determine factors affecting survival rates, benefits and complications of patients with obstructive esophageal cancer treated with photodynamic therapy (PDT).

*Methods* From 1982 to January 1998, we used PDT to treat 140 patients with obstructive adeno or squamous carcinoma and evaluated survival up to November 1998. All patients had failed, refused, or were ineligible for surgery, ionizing radiation or chemotherapy. The effect of different variables on survival was estimated using multivariate analysis. The Karnofsky Performance Status (KPS), weight, diet and complications were recorded and biopsies and brushings were taken at each endoscopy. At the beginning and end of each endoscopy the minimal diameter open of the esophagus, and the length, thickness and color of the tumor were recorded. Edema, exudate, bleeding, and mucositis were evaluated and recorded on an ordinal scale.

*Results* The only significant variable affecting survival was the clinical stage. The median survival after PDT for all patients was 6.5 months (mean = 13.9). Kaplan–Meier survival after PDT curves were statistically significantly different when stratified by the clinical Stage at the time of PDT (*p* < 0.0001). Median survival (months) were for: Stage I  = 56; Stage II  = 12; Stage III = 6.5; Stage IV = 3.5. Analysis of each individual stage showed the KPS was the only confounding variable with a statistically significant effect on survival after PDT and this was only for Stages III and IV. The most significant effect occurred when the KPS was ≥ 70. For Stage III the median survival when the KPS was ≥ 70 was 7.7 months and for a KPS < 70 it was 5.0 months (*p*  = 0.0001). For Stage IV the median survival when the KPS was ≥ 70 was 5.5 months and for a KPS < 70 it was 2.5 months (*p*  = 0.0002). The mean minimum diameter open before PDT was 6.2 mm (median 6.0mm) and at the end of the PDT treatment endoscopy 11.1 mm (median 12.0 mm) for a mean increase in the minimum diameter open of 4.9 mm (median 5.0 mm) This was statistically significant using paired *t*-tests (*p* < 0.0001).

*Conclusions* Photodynamic therapy for esophageal carcinoma caused minimal complications and procedure related mortality. Complete obstruction can be relieved by the end of the PDT endoscopy. The length of palliation for “non-curative” patients was equal to or better than that reported historically for most other treatment regimens.


*Diagnostic and Therapeutic Endoscopy*, Vol. 5, pp. 167–174
Reprints available directly from the publisher
Photocopying permitted by license only

© 1999 OPA (Overseas Publishers Association) N.V.
Published by license under
the Harwood Academic Publishers imprint,
part of The Gordon and Breach Publishing Group.
Printed in Malaysia.

### Photodynamic Therapy for Obstructive Esophageal Malignancies

JAMES S. McCAUGHAN, JR.*

*Grant Medical Center, 323 E. Town Street, Columbus, OH 43215, USA*

***Objectives*** **Determine factors affecting survival rates, benefits and complications of patients with obstructive esophageal cancer treated with photodynamic therapy (PDT).**

***Methods*** **From 1982 to January 1998, we used PDT to treat 140 patients with obstructive adeno or squamous carcinoma and evaluated survival up to November 1998. All patients had failed, refused, or were ineligible for surgery, ionizing radiation or chemotherapy. The effect of different variables on survival was estimated using multivariate analysis. The Karnofsky Performance Status (KPS), weight, diet and complications were recorded and biopsies and brushings were taken at each endoscopy. At the beginning and end of each endoscopy the minimal diameter open of the esophagus, and the length, thickness and color of the tumor were recorded. Edema, exudate, bleeding, and mucositis were evaluated and recorded on an ordinal scale.**

***Results*** **The only significant variable affecting survival was the clinical stage. The median survival after PDT for all patients was 6.5 months (mean = 13.9). Kaplan–Meier survival after PDT curves were statistically significantly different when stratified by the clinical Stage at the time of PDT ($p < 0.0001$). Median survival (months) were for: Stage I = 56; Stage II = 12; Stage III = 6.5; Stage IV = 3.5. Analysis of each individual stage showed the KPS was the only confounding variable with a statistically significant effect on survival after PDT and this was only for Stages III and IV. The most significant effect occurred when the KPS was $\geq 70$. For Stage III the median survival when the KPS was $\geq 70$ was 7.7 months and for a KPS $< 70$ it was 5.0 months ($p = 0.0001$). For Stage IV the median survival when the KPS was $\geq 70$ was 5.5 months and for a KPS $< 70$ it was 2.5 months ($p = 0.0002$). The mean minimum diameter open before PDT was 6.2 mm (median 6.0 mm) and at the end of the PDT treatment endoscopy 11.1 mm (median 12.0 mm) for a mean increase in the minimum diameter open of 4.9 mm (median 5.0 mm) This was statistically significant using paired *t*-tests ($p < 0.0001$).**

***Conclusions*** **Photodynamic therapy for esophageal carcinoma caused minimal complications and procedure related mortality. Complete obstruction can be relieved by the end of the PDT endoscopy. The length of palliation for "non-curative" patients was equal to or better than that reported historically for most other treatment regimens.**

*Keywords:* Esophageal cancer, Photodynamic therapy, Survival

* Tel.: (614)221-2643.

*Abbreviations:* PDT, photodynamic therapy; KPS, Karnofsky performance status; DHE, dihematoporphyrin ether; LMB, left main bronchus

### INTRODUCTION

The use of selective photodynamic therapy (PDT) to treat malignant tumors is based on three observations: (1) after being injected intravenously, the photosensitizer disseminates to all cells; (2) due to differences in vascular and lymphatic clearance from tumors, and retention of the photosensitizer by the tumor cells, the photosensitizer is selectively retained in the tumor cells and interstitial tissue of the tumor so that after two or three days there is a greater concentration of the photosensitizer in the tumors than in the adjacent normal tissue; (3) the photosensitizer will absorb light energy and produce singlet oxygen which then destroys the tumor. Since there is less photosensitizer in the adjacent tissue, it will react less. We report here our experience using PDT to treat patients with obstructive esophageal cancer.

### MATERIALS AND METHODS

In a prospective study from 1982 to January 1998 we used PDT to treat 140 patients with obstructive esophageal carcinoma and evaluated their survival up to November 1998. Entry criteria were a histologic diagnosis of adeno or squamous cell carcinoma of the esophagus and failure of conventional treatment or ineligibility for them because of medical status. There was 100% follow up.

Their ages ranged from 35 to 91 with a mean of 68 and median of 67 years. One hundred and forty patients (95 adenocarcinoma and 45 squamous) had varying degrees of obstruction and difficulty swallowing with a mean minimum diameter of 6.2 mm (median 6.0 mm).

All patients were clinically staged at the time of their PDT using the TNM system [1] based on the history, physical examination, bronchoscopy, esophagoscopy, barium esophagrams and CT scans of the chest and abdomen. Fourteen were Stage I (13 adeno, 1 squamous), 23 were Stage II (18 adeno, 5 squamous), 51 were Stage III (28 adeno, 23 squamous), and 52 were Stage IV (36 adeno, 16 squamous).

The Karnofsky Performance Status (KPS) [2], weight, diet and complications were recorded and biopsies and brushings were taken at each endoscopy. At the beginning and end of each endoscopy the minimal diameter open of the esophagus, and the length, thickness and color of the tumor were recorded. Edema, exudate, bleeding, and mucositis were evaluated and recorded on an ordinal scale.

Photodynamic therapy was performed using 630 nm light generated by either an argon dye laser system (Spectra Physics) or a double frequency YAG-dye laser system (Laserscope) as the activator and delivered through cylinder diffusing tip quartz fibers passed through the biopsy channel of a flexible endoscope. When possible the diffuser tip was inserted into the tumor. Otherwise, it was placed along side of the tumor.

Initial treatments were performed using hematoporphyrin derivative as the photosensitizer but for the last 14 years we have used dihematoporphyrin ether (DHE, Photofrin; Quadra Logic Technologies, Vancouver, B.C.) injected intravenously one to three days before the treatment.

Various photosensitizer doses, day of treatment after injection, light power densities, and light doses were evaluated [3–8]. Analyses of the results from these various parameters lead us to now use 60 mg of DHE per meter squared of body surface [9]; power densities of 400 mW per centimeter of diffusing fiber; light doses of 300 J per centimeter of diffusing fiber, interstitial treatment of the tumor if possible, and treatment 1–3 days after injection of the photosensitizer.

Two to three days after PDT, esophagoscopy was repeated and necrotic tissue mechanically removed.

One month after PDT a repeat endoscopy was done and residual tumor was treated following another injection of photosensitizer. Patients were periodically endoscoped and retreated for symptomatic residual tumor.

### Statistical Analyses

Statistical analyses were performed using SuperANOVA, StatView, and Survival Tools (Abacus Concepts, Inc., Berkeley, CA). All statistics used 95% confidence limits. Disease specific survival times in months were calculated from the time of their first PDT to the end point of November 1998 using Kaplan–Meier tables and curves. The Breslow–Gehan–Wilcoxon test was used to compare the significance of differences of survival distributions because it is more likely to detect early differences than logrank tests and most of the deaths occur early in all series of esophageal cancer.

Cox proportional hazards tests were used to estimate the effects of different variables on the length of survival.

Paired $t$-tests were used to evaluate the increase in the minimum diameter open at the end of the PDT endoscopy.

### RESULTS

### Survival

The effect of different variables on survival of 140 patients with obstructive adeno or squamous carcinoma after PDT was estimated using multivariate analysis. A model of the effects of age, sex, race, KPS, day of treatment, histology, location of tumor, length of tumor, minimum diameter of esophagus open and clinical Stage showed the only statistically significant variable was the Stage ($p = 0.0008$). The Global likelihood ratio for the model was significant ($p < 0.0001$).

The median survival after PDT for all patients was 6.5 months (mean = 13.9). Kaplan–Meier survival after PDT curves were statistically significantly different when stratified by the clinical Stage at the time of PDT ($p < 0.0001$). Median survival (months) were for: Stage I = 56; Stage II = 12; Stage III = 6.5; Stage IV = 3.5 (Fig. 1).

Analysis of each individual stage showed the KPS was the only confounding variable with a statistically significant effect on survival after PDT, but only for Stages III and IV. The most significant effect occurred when the KPS was $\geq 70$. For Stage III the median survival when the KPS was $\geq 70$ was 7.7 months and for a KPS $< 70$ it was 5.0 months ($p = 0.0001$). For Stage IV the median survival when the KPS was $\geq 70$ was 5.5 months and for a KPS $< 70$ it was 2.5 months ($p = 0.0002$).

### Increase in Diameter Open at End of PDT

The mean minimum diameter open before PDT was 6.2 mm (median 6.0 mm) and at the end of the PDT treatment endoscopy 11.1 mm (median 12.0 mm) for a mean increase in the minimum diameter open of 4.9 mm (median 5.0 mm). This was statistically significant using paired $t$-tests ($p < 0.0001$).

### Complete Obstruction of Esophagus

Twenty-five patients were completely obstructed. The mean diameter open before PDT was 0.89 mm (median 0 mm) and at the end of PDT the mean and median diameter open were 10.0 mm (Fig. 2).

### Complications

Transient elevations of the WBC and temperature frequently developed immediately after PDT. Unilateral or bilateral pleural effusions may occur over several days but these spontaneously resolved.

Four patients had pulmonary complications after PDT (infiltrates 2, aspiration pneumonia 1, pulmonary edema 1; all resolved).

Four patients developed fistulae related to PDT (two trachea, two LMB). All had squamous cell cancer. One had visible tumor in the LMB before PDT. Two were Stage III and two Stage IV. One died from GI bleeding after insertion of an esophageal stent. The other three died of their

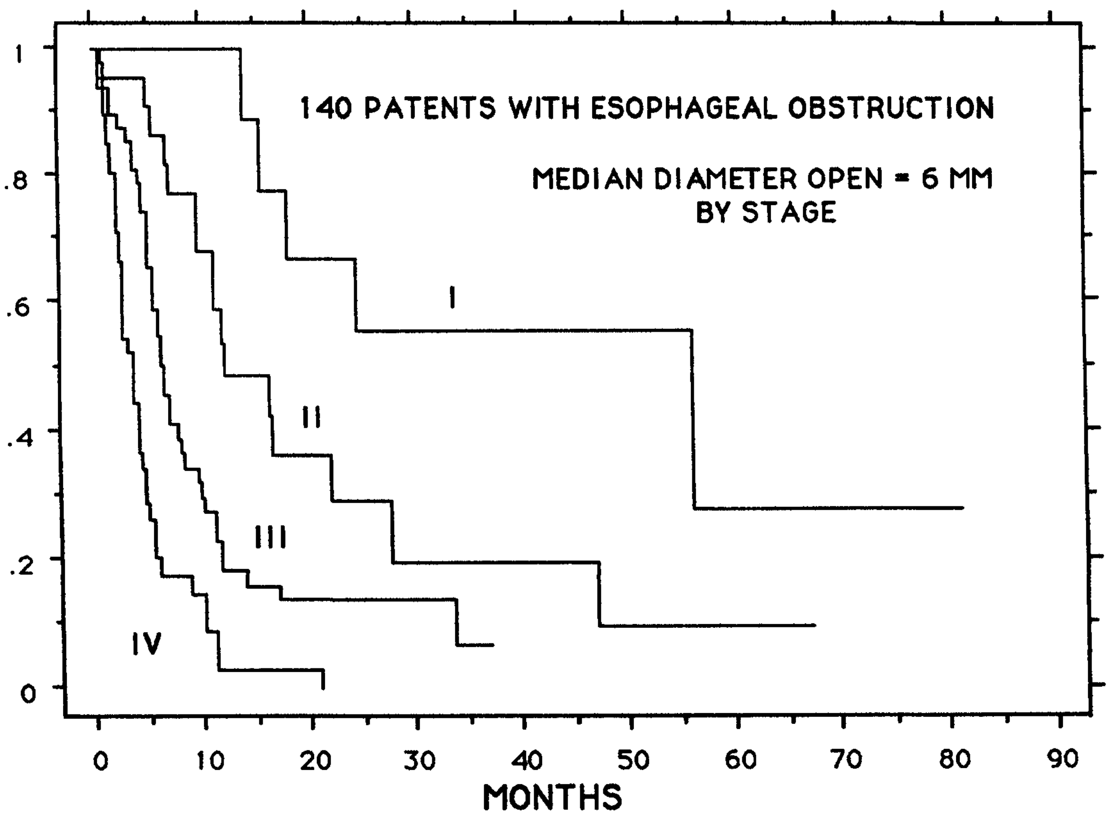

FIGURE 1 Kaplan–Meier cumulative survival curves for patients with obstructive carcinoma of the esophagus treated with photodynamic therapy stratified by their clinical stage at the time of PDT.

disease. Treatment before PDT consisted of: external radiation to one; brachytherapy and YAG laser to one; brachytherapy, chemotherapy and immunotherapy to one; and external radiation, chemotherapy and YAG laser to one.

Four patients developed strictures after PDT. All were manageable with dilation.

Solar photosensitivity of the skin may last for up to 8 weeks after the injection of DHE. Five patients developed erythema and itching on the hands and/or face from sun exposure. They all resolved spontaneously. Three patients developed edema of the hands and one of the face. These completely resolved over a few days with oral steroids. With careful increasing sun exposure and repeated instructions, there have not been any serious photosensitivity reactions to the sun.

### DISCUSSION

The most significant variable affecting the length of survival in this series was the stage of the disease. When staged, there was no statistically significant difference due to age, sex, race, day of treatment, length of tumor, minimal diameter of esophagus open, or location of tumor. There was no statistically significance difference in the survival distributions within each stage between adenocarcinoma and squamous cell carcinoma. However, within Stages III and IV a KPS above or equal to 70 was a significant favorable prognosticator and should be considered in comparing survival rates from different treatment regimens for these stages.

Photodynamic therapy for esophageal carcinoma caused minimal complications and procedure

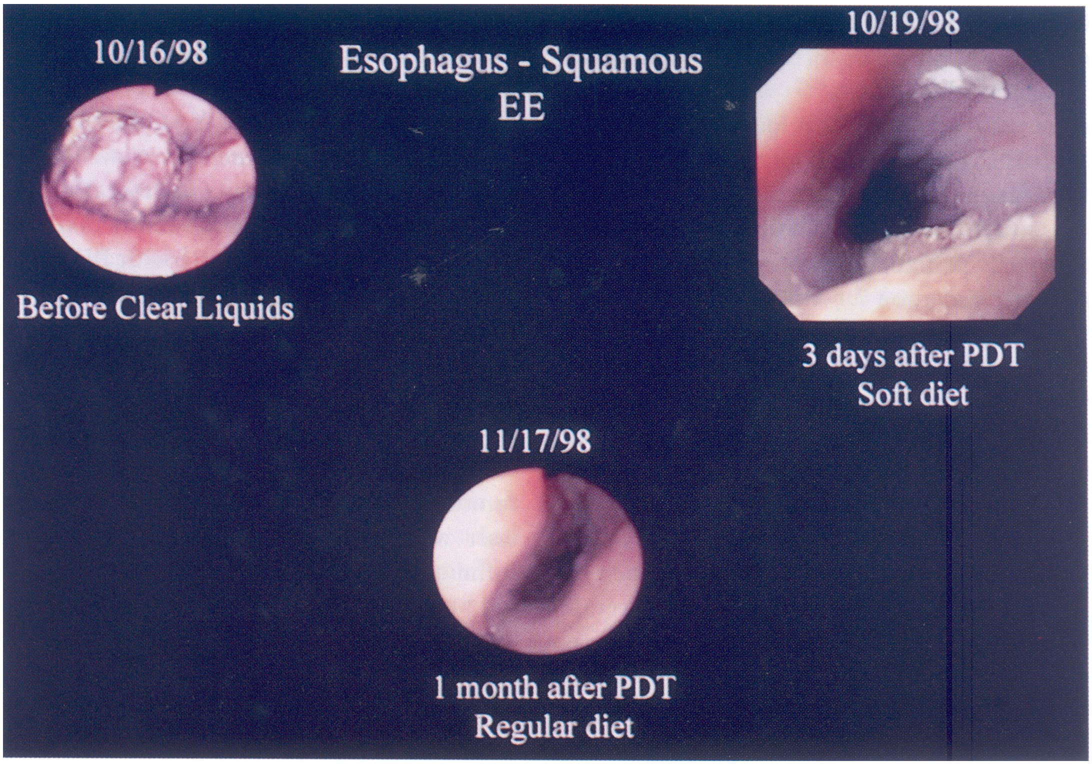

FIGURE 2 Complete obstruction of esophagus by squamous cell carcinoma opened at the end of PDT endoscopy and one month after PDT.

related mortality. The duration of palliation for "non-curative" patients was equal to or better than that reported historically for most other treatment regimens [10].

### Current Technique

We use general endotracheal anesthesia with a 7.5 mm endotracheal tube. After intubation the patient is put in high semi-Fowler's position and continuous oral suction is used to minimize aspiration. If needed we use teflon guide wires and an image intensifier to guide the scope or for use during dilation with Savory or balloon dilators. PDT is performed interstitially when possible. It is better to under-treat than over-treat. Surface PDT is done retrogradely using 2.5 cm diffusing tips. The tip is moved back 3 cm to perform successive treatments to minimize overlap. Although 300 J/CF is used for completely circumferential tumors, we use only 200 J/F if it appears non-tumor mucosa will be exposed to the light, such as at the ends of the tumor.

A repeat esophagoscopy and dilation is done two days after PDT but we rarely retreat at this time. Patients are rescoped one month after PDT and we may retreat at this time *without* another injection [11]. At this time we use 300 J/CF even on the "normal" mucosa.

### Comparison of Survival from other Treatment Regimens

In a retrospective analysis of survival from diagnosis of 268 patients with carcinoma of the esophagus Oliver *et al.* [12] found the overall median survival (months) stratified by primary treatment was: surgery = 9.8; radical X-ray = 6.3; intubation = 3.3; palliative radiotherapy = 2.7 months. Unfortunately they were not staged but only 34% were eligible for surgery with an operative mortality of 9%. Intubation had a mortality of 15%.

Fok *et al.* [13] compared patients that had radiation treatment after surgery with those who only had surgery. The median survivals (months) were: curative resection 21; curative resection with radiation 15; palliative resection 12; palliative resection with radiation 7.

Urba *et al.* [14] reported a median survival of 11 months for 24 patients with adenocarcinoma of the esophagus treated with preoperative chemotherapy and radiation. Twenty-two were Stage II, one Stage I and one Stage III.

Poplin *et al.* [15] reported on 106 patients with squamous cell carcinoma of the esophagus registered into a study combining preoperative chemotherapy and external radiation with an overall median survival of 12 months and an 11% operative mortality.

LePrise *et al.* [16] reported preoperative chemotherapy and radiation (CRT) did not change the operative mortality or survival time for patients with Stages I and II squamous cell carcinoma of the esophagus. The operative mortality was 8.5% for CRT and 7% for without CRT. The median survival was 10 months for both groups.

Skinner *et al.* [17] reported the median survival of those patients chosen for "standard esophagectomy" for palliation because of extensive disease was 8 months.

Law *et al.* [18] reported median survivals after palliative resection of 7 months for 236 patients with squamous cell and 8 months for 57 patients with adenocarcinoma.

We also treated 33 patients with the YAG laser for esophageal obstruction [8] and compared our results with PDT. PDT is easier to perform than YAG laser treatments. There is less possibility of burning a hole in the tissue with PDT. On occasion, it is impossible to get the YAG laser in position to safely treat the tumor with the YAG because of the anatomy. Cervical lesions are difficult to treat with the YAG laser because one may not be able to keep the endoscope in the esophagus. The PDT diffuser tip can be inserted into or along side of the tumor and the treatment performed blindly. Blind treatment with the YAG laser invites disaster. It is difficult to treat the tumors with the YAG laser antegradely because the charred, burned tissue swells and makes it difficult to find the correct path for distal lasering. Completely obstructed hard tumors that cannot be dilated can be treated blindly with PDT, and they soften by the end of treatment. PDT also treats submucosal spread that is not visible and can be used to treat small mammary-like tumors, whereas the YAG might burn through the esophagus. Exophytic lesions that are large or very bloody are better treated with the YAG. Debulking large tumors and treating the residual tumor a few weeks later with PDT utilizes the advantages of both techniques [19].

### Barrett's Dysplasia and Early Stage Adenocarcinoma

The incidence of adenocarcinoma of the esophagus and Barrett's esophagus has been increasing since the early 1980s [20–24].

At the same time the management of reflux esophagitis changed dramatically with introduction of histamine $H_2$ receptor antagonists – in 1977 cimetidine (Tagamet), 1983 ranitidine hydrochloride (Zantac), and 1986 famotidine (Pepsid). Gastric acid pump inhibitors were then approved – in 1989 omeprazole (Prilosec) and 1995 lansoprazole (Prevacid). Prior to these drugs, patients with symptomatic hiatal hernias had them surgically repaired but with the introduction of these medications the number of operations markedly decreased. Medical management may relieve the symptoms, but it does not correct the reflux nor cause the Barrett's metaplasia to return to normal squamous mucosa. Once Barrett's metaplasia has started, the reflux repair and medications will not stop the development of the carcinoma. Most adenocarcinomas of the esophagus arise in Barrett's metaplasia [21,23,25,26]. Most Barrett's are caused by acid reflux [27,28]. Most reflux is due to hiatal hernias [29–32].

Overholt and Panjehpour [33] reported a 75–80% reduction of dysplasia in 36 patients with Barrett's treated with PDT with DHE. Ten had complete replacement with squamous epithelium and 14 patients with stage I cancer had complete destruction of their tumors.

After PDT, the Barrett's mucosa becomes necrotic and is replaced with squamous epithelium. However, two of our patients with stage I adenocarcinoma associated with Barrett's metaplasia and hiatal hernia had a complete response and normal squamous epithelium 3 months after PDT, but developed recurrence of the Barrett's mucosa at 10 and 15 months, in spite of medical management with omeprazole. The hiatal hernias were not repaired. Biddlestone *et al.* [34] found histologic residual glandular mucosa, non-neoplastic and dysplastic, beneath squamous epithelium after acid suppression and laser and photodynamic therapy indicating the requirement for histologic confirmation of endoscopically suspected complete squamous re-epithelialization with sufficiently deep biopsies. Advantages of PDT for Barrett's are: no procedure related mortalities, it can be repeated indefinitely, and the survival rate appears to be as good as surgery.

We suggest: Symptomatic hiatal hernia without Barrett's should be surgically repaired. Current Barrett's metaplasia should be treated with PDT to destroy the Barrett's and the hiatal should be repaired. Patients should be followed with endoscopy and medical management until it is established there is no residual or recurrent esophagitis. Any residual Barrett's should be retreated with PDT.

### CONCLUSIONS

The only statistically significant variable affecting survival after PDT to carcinoma of the esophagus was the clinical stage. Analysis of each individual stage showed the KPS was the only statistically significant confounding variable and the most significant effect occurred when the KPS was $\geq 70$ for Stages III and IV. Completely obstructed hard tumors that cannot be dilated can be treated blindly with PDT, and they soften by the end of treatment. Cervical lesions are difficult to treat with the YAG laser while the PDT diffuser tip can be inserted and the treatment performed blindly. PDT also treats submucosal spread that is not visible and can be used to treat small mammary-like tumors. Debulking large tumors and treating the residual tumor with PDT utilizes the advantages of both techniques. Palliation with PDT compares favorably to surgery for "non-curative" disease. PDT can be used concomitantly with chemotherapy and X-ray irradiation and can be repeated indefinitely. Photosensitivity of the skin to solar irradiation due to the photosensitizer is more of a nuisance than a serious problem for most of these patients.

### *References*

[1] Beahrs, O.H. *et al.* Eds. *Manual for Staging of Cancer*, 4th Ed. Am. Joint Committee on Cancer, Phila: JB Lippincott Co. 1992.

[2] Karnofsky, D.A. and Burchenal, J.H. The clinical evaluation of chemotherapeutic agents in cancer. In Macleod C.M., Ed. *Evaluation of Chemotherapeutic Agents.* New York: Columbia University Press, 1949; 199–205.

[3] McCaughan, J.S. Jr., Hicks, W., Laufman, L., May, E. and Roach, R. Palliation of esophageal malignancy with photoradiation therapy. *Cancer* 1984; **54**: 2905–2910.

[4] McCaughan, J.S. Jr., Williams, T.E. Jr. and Bethel, B.H. Palliation of esophageal malignancy with Photodynamic therapy. *Ann. Thor. Surg.* 1985; **40**: 113–120.

[5] McCaughan, J.S. Jr., Nims, T.A., Guy, J.T., Hicks, W.J., Williams, T.E. Jr. and Laufman, L.R. Photodynamic therapy for esophageal tumors. *Arch. Surg.* 1989; **124**: 74–80.

[6] McCaughan, J.S. Jr., Barabash, R.D., Penn, G.M. and Glavan, B.J. Nd : Yag laser and photodynamic therapy for esophageal and endobronchial tumors under general and local anesthesia. Effects on arterial blood gas levels. *Chest* 1990; **98**: 1374–1378.

[7] McCaughan, J.S. Jr. Photodynamic therapy of skin and esophageal cancers. *Cancer Investigation* 1990; **8**(3/4): 407–416.

[8] McCaughan, J.S. Jr. Photodynamic therapy versus Nd-Yag laser treatment of endobronchial or esophageal malignancies. In: Spinelli, P., Dal Fante, M. and Marchesini, R. (Eds.) *Photodynamic Therapy and Biomedical Lasers.* Elsevier Science Publishers 1992; 23–36.

[9] McCaughan, J.S. Jr. and Miller, C. "Photosensitizer dosage: mg/kg or mg/2?" Optical Methods for Tumor Treatment and Detection: Mechanisms and Techniques In: Thomas, J. Dougherty (Ed.) *Photodynamic Therapy II, Proc. SPIE.* 1993; **1881**: 26–34.

[10] McCaugen, J.S. Jr., Ellison, E.C., Guy, J.T. *et al.* Photodynamic therapy for esophageal malignancy: a prospective 12 year study. *Ann. Thorac. Surg.* 1996; **62**: 1005–1010.

[11] McCaughan, J.S. PDT of endobronchial tumors on the same day as injection of the photosensitizer DHE. *Clinical Laser Monthly April* 1993; 57–58.
[12] Oliver, S.E., Robertson, C.S. and Logan, R.F.A. Oesophageal cancer: a population-based study of survival after treatment. *Br. J. Surg.* 1992; **79**: 1321–1325.
[13] Fok, M., Sham, J.S., Choy, D., Cheng, S.W.K. and Wong, J. Postoperative radiotherapy for carcinoma of the esophagus: a prospective, randomized controlled study. *Surgery* 1993; **113**: 138–147.
[14] Urba, S.G., Orringer, M.B., Perez-Tamayo, C., Bromberg, J. and Forastierre, A. Concurrent preoperative chemotherapy and radiation therapy in localized esophageal adenocarcinoma. *Cancer* 1992; **69**: 285–291.
[15] Poplin, E., Fleming, T., Leichman, L., Seydel, H.G., Stefger, S., Taylor, S., Vance, R., Stuckey, W.J. and Rivkin, S.E. Combined therapies of squamous-cell carcinoma of the esophagus: a Southwest Oncology Group study. *J. Clin. Oncol.* 1987; **5**; 622–628.
[16] LePrise, E., Etienne, P.L., Meunier, B., Maddern, G., Ban Hassel, M., Gedouin, D., Boutin, D., Campion, J.P. and Launois, B. A randomized study of chemotherapy, radiation therapy, and surgery for localized squamous cell carcinoma of the esophagus. *Cancer* 1994; **73**: 1779–1784.
[17] Skinner, D.B., Ferguson, M.K., Soriano, A. and Little, A.G. Selection of operation for esophageal cancer based on staging. *Ann. Surg.* 1986; **204**: 391–401.
[18] Law, S.Y.K., Fok, M., Cheng, S.W.K. and Wong, K. A comparison of outcome after resection for squamous cell carcinomas and adenocarcinomas of the esophagus and cardia. *Surg. Gynecol. Obstet.* 1992; **175**: 107–112.
[19] McCaughan, J.S. Jr. Photodynamic therapy versus Nd-Yag Laser Treatment of Endobronchial or Esophageal Malignancies. In *Photodynamic Therapy and Biomedical Lasers.* Spinelli, P., Dal Fante, M. and Marchesini, R. (Eds.) Elsevier Science Publishers, 1992; 23–36.
[20] Hesketh, P.J., Clapp, R.W., Doos, W.G.S. *et al.* The increasing frequency of adenocarcinoma of the esophagus. *Cancer* 1989; **64**: 526–530.
[21] Blot, W.J., Devesa, S.S., Kneller, R.W. *et al.* Rising incidence of adenocarcinoma of the esophagus and gastric cardia. *JAMA* 1991; **265**: 1287.
[22] Birgisson, S., Rice, T.W., Kirk, A. *et al.* The lack of association between adenocarcinoma of the esophagus and gastric surgery: a retrospective study. *Am. J. Gastroenterol.* 1997; **92**: 216–221.
[23] Kirby, T.J. and Rice, T.W. The epidemiology of Esophageal carcinoma In: Rice, T.W. and Kirby, T.J. (Eds.) *Chest Surgery Clinics of North America. Esophageal Carcinoma* Phila: WB Saunders Pub 1994; **4**(2): 217–225.
[24] Hansen, S., Wilg, J.N., Glercksky, K.E. *et al.* Esophageal and gastric carcinoma in Norway 1958–1992: incidence time trend variability according to morphological subtypes and organ subsites. *Int. J. Cancer* 1997; **71**: 340–344.
[25] Reld, B.J. Barrett's esophagus and esophageal adenocarcinoma. Mucosal diseases of the esophagus. *Gastroenterol. Clin. North Am.* 1991; **20**: 817–834.
[26] Clark, G.W., Smyrk, T.C., Burdiles, P. *et al.* Is Barrett's metaplasia the source of adenocarcinoma of the cardia? *Arch. Surg.* 1994; **129**: 609–614.
[27] Ollyo, J.B., Monnter, P., Fontolliet, C. *et al.* The natural history, prevalence and incidence of reflux oesophagitis. *Gullet* 1993; Sup. 3; 3–10.
[28] Orlando, R.C. Reflux esophagitis in Textbook of Gastroenterology. In: Yamada, T. *et al.* (Eds.) 2nd edn. Pub. Phila: JB Lippincott. 1995; 1214–1242.
[29] Criman, D.K. and Riddell, R.H. The distinction between adenocarcinoma of the cardia and Barrett' cancer. pp. 236–253. In: Wastell, C., Nyhus, L.M. and Donahue, P.E. (Eds.) *Surgery of the Esophagus, Stomach and Small Intestine*, Boston: Little, Brown and Company, 1995.
[30] Phillips, R.W. and Wong, R.K.H. Barrett's esophagus: natural history, incidence, etiology and complications. *Gastroenterol. Clin. North. Am.* 1991; **20**: 791.
[31] Ott, D.J., Wu, W.C. and Gelfand, D.W. Reflux esophagitis revisited: prospective analysis of radiological accuracy. *Gastrointest. Radiol.* 1981; **6**: 1.
[32] Kerlin, P., d'Mellow, G. and van Deth, A. Barrett's esophagus: clinical, endoscopic and histologic spectrum in fifty patients. *Aust. N Z. J. Med.* 1986; **16**: 198–205.
[33] Overholt, B.F. and Panjehpour, M. Photodynamic therapy for Barrett's esophagus: clinical update. *Am. J. Gastroenterol.* 1996; **91**(9): 1719–1723.
[34] Biddlestone, L.R., Barham, C.P., Wilkinson, S.P. *et al.* The histopathology of treated Barrett's esophagus: squamous reepithelization after acid suppression and laser and photodynamic therapy. *Am. J. Surg. Pathol.* 1998; **22**(2): 239–245.

